# Management of Phlegmasia Cerulea Dolens Alongside Neurologic Contraindication to Anticoagulation: A Therapeutic and Diagnostic Dilemma

**DOI:** 10.1002/ccr3.71175

**Published:** 2025-11-10

**Authors:** Reem Sarsour, Veerpal Sond, Cameron Casillas, Elizabeth Morrison‐Banks, Niki Mohammadi

**Affiliations:** ^1^ Department of Internal Medicine Arrowhead Regional Medical Center Colton California USA

**Keywords:** anticoagulation, cerebrovascular, deep vein thrombosis, thrombectomy, vascular surgery

## Abstract

The purpose of this case report is to highlight anticoagulation management of phlegmasia cerulea dolens, particularly in a patient with a recent ischemic stroke that underwent hemorrhagic transformation. In select cases, delayed anticoagulation after appropriate neurological clearance can be a viable approach to prevent thrombotic progression while minimizing hemorrhagic risk.

## 1 Introduction

Phlegmasia Cerulea Dolens (PCD) is a rare and life‐threatening vascular condition characterized by massive deep vein thrombosis (DVT) of the limb leading to catastrophic venous obstruction and severe venous congestion [1]. Latin for “painful blue inflammation,” PCD is considered one of the most severe forms on the DVT spectrum, with high rates of limb ischemia, amputation, and mortality [2]. Its occurrence is so uncommon that estimates regarding incidence cannot be reliably predicted [2]. PCD involves near‐total occlusion of the major deep venous system and a substantial proportion of the collateral microvasculature of the extremity [2]. Clinical manifestations include a triad of pain, swelling, and cyanosis in the affected limb [2]. Etiologies include venous stasis and hypercoagulability from immobility, malignancy, heart failure, pregnancy, or antiphospholipid syndrome [2]. This case report contributes to the limited literature on PCD and proposes a treatment pathway tailored for patients with contraindications to anticoagulation. It offers practical guidance for physicians managing similar clinical dilemmas.

## 2 Case History

A 52‐year‐old male presented to the emergency department with a 4‐h history of sudden‐onset painful blue discoloration of his left leg. Four weeks earlier, he sustained an ischemic stroke complicated by hemorrhagic conversion one week later, resulting in residual left‐sided paralysis. A week after the hemorrhagic stroke, he developed his first episode of venous thromboembolism with DVT and multiple pulmonary emboli. The immobility following the stroke contributed to this initial event. He was not treated with anticoagulation due to the recent intracranial hemorrhage; his only intervention was placement of an inferior vena cava (IVC) filter. This episode of PCD was precipitated by the inadequately treated DVT, which could not be managed with anticoagulation because of his hemorrhagic stroke. His ongoing risk factors for thrombosis included stroke‐related immobility exacerbated by delays in physical therapy, venous stasis, and a 40‐year smoking history.

**FIGURE 1:**
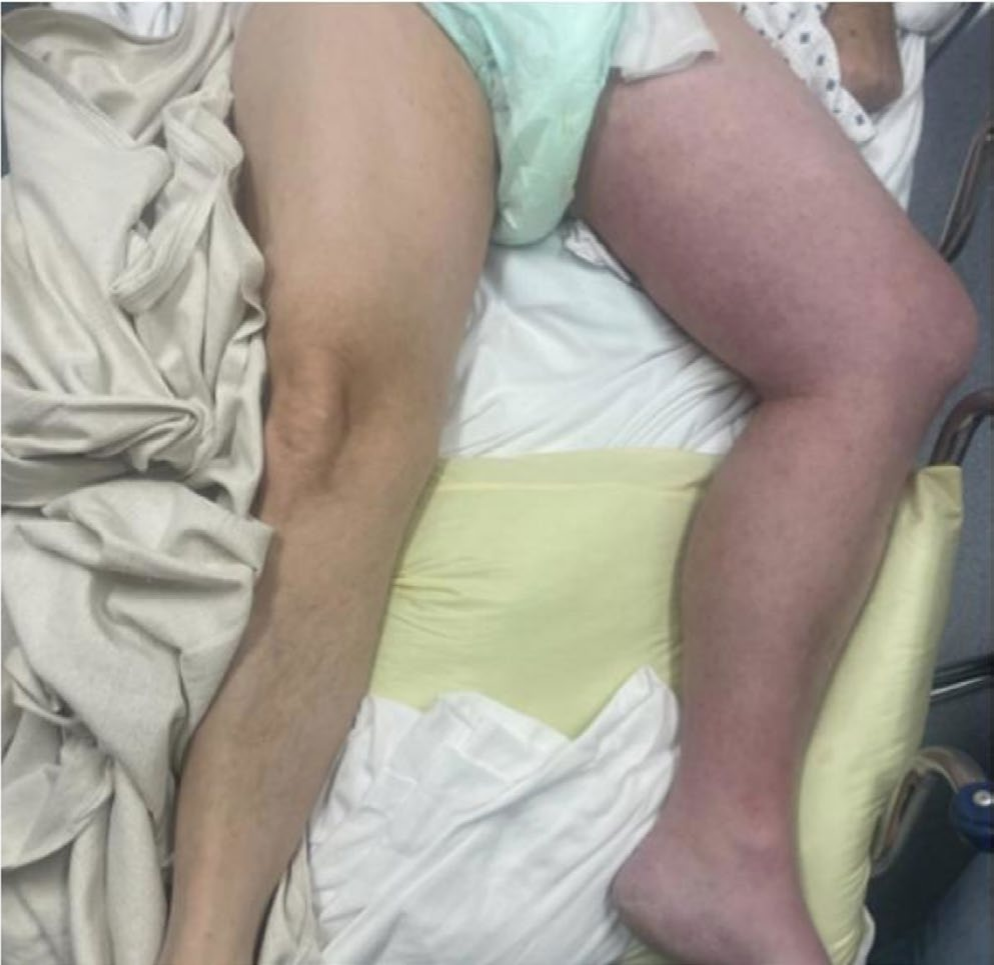
Emergency department presentation of left leg. Swelling and circumferential cyanotic discoloration extending from mid‐left foot to left proximal thigh near the region of inguinal ligament.

In the emergency department, he reported severe pulsating pain radiating from the left leg into the suprapubic region, abdomen, and lower back. He was tachycardic, with labs showing mild leukocytosis (Tables 1 and 2). On exam, the left leg demonstrated red‐blue discoloration (Figure 1). The left leg was cold to touch and had diminished but palpable dorsalis pedis, femoral, and popliteal pulses (+1). A CT chest with contrast confirmed the IVC filter, with a breakthrough nonocclusive PE in the right interlobar artery. The CT chest did not extend caudally to fully evaluate the infrarenal IVC filter for thrombosis; thus, in‐filter thrombus could not be excluded. Evaluation of the filter was deferred to the outpatient setting, as the immediate priority was acute PCD management.

**TABLE 1:** Emergency department triage vitals of patient upon admission.

Emergency department triage vitals	
Heart rate	116
Respiratory rate	20
Blood pressure	93/68
SpO_2_	93%

**TABLE 2:** Laboratory values of complete blood count of patient upon admission in the emergency department.

Complete blood count in emergency department	
White blood cells	12.8
Hemoglobin	14.3
Hematocrit	41
Platelets	230

## 3 Differential Diagnosis, Investigations and Treatment

**FIGURE 2:**
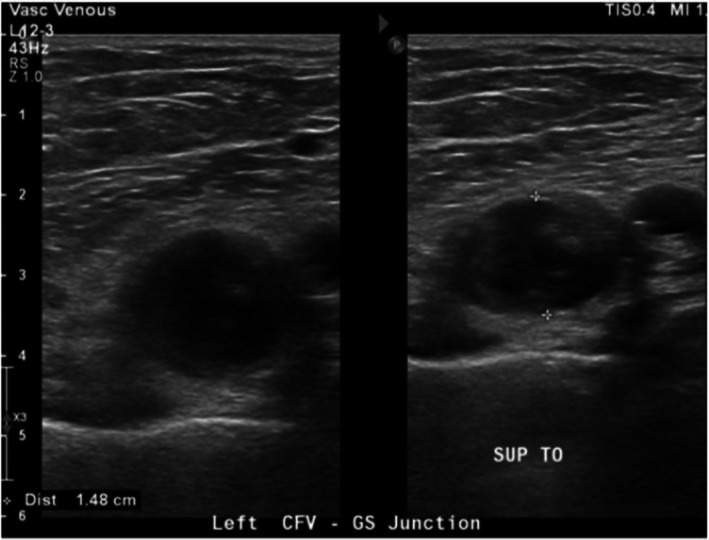
Duplex ultrasound of the left common femoral vein (CFV) at the greater saphenous (GS) junction. Transverse view demonstrating a noncompressible, echogenic thrombus within the CFV with absent venous collapse on probe compression, consistent with acute DVT. The crosshair calipers delineate the thrombus within the lumen.

Given the marked discoloration, diminished pulses, and severe pain, it was essential to promptly exclude arterial occlusion, as delayed recognition of acute limb ischemia carries a high risk of compartment syndrome and amputation. Vascular surgery performed a duplex ultrasound of bilateral lower extremities. On the left, an incompressible, distended echogenic thrombus with minimal flow was seen at the common femoral–greater saphenous vein junction (Figure 2). This ruled out acute arterial ischemia and redirected management toward PCD.

**FIGURE 3:**
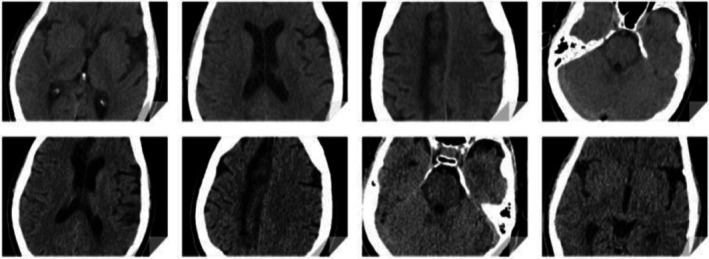
Axial CT head non‐contrast slices displaying brain images taken within 12 h of admission (top row) and approximately 24 h after initiating heparin therapy (bottom row). Axial CT slices demonstrate prior ischemic infarcts with small areas of hemorrhagic conversion (hyperdense foci) without evidence of new acute intracranial hemorrhage.

A non‐contrast CT head was considered to evaluate for acute hemorrhagic changes after his prior hemorrhagic stroke (Figure 3). Since a contrast CT chest had already been obtained for PE, the CT head was delayed 12 h to allow for contrast clearance. Neurology and neurosurgery emphasized obtaining CT head imaging before starting anticoagulation. During this period, conservative management with leg elevation, analgesia, and loose elastic wrapping was used. Twelve hours later, the non‐contrast CT head showed no acute changes. Given the massive DVT, stable neuroimaging, and no new neurological deficits, intravenous unfractionated heparin was initiated without a bolus, titrated to therapeutic aPTT. Neuro checks with pupillometry were performed every 4 h, and a repeat CT head was obtained 24 h later at therapeutic heparin levels (Figure 3).

## 4 Conclusion and Results

**FIGURE 4:**
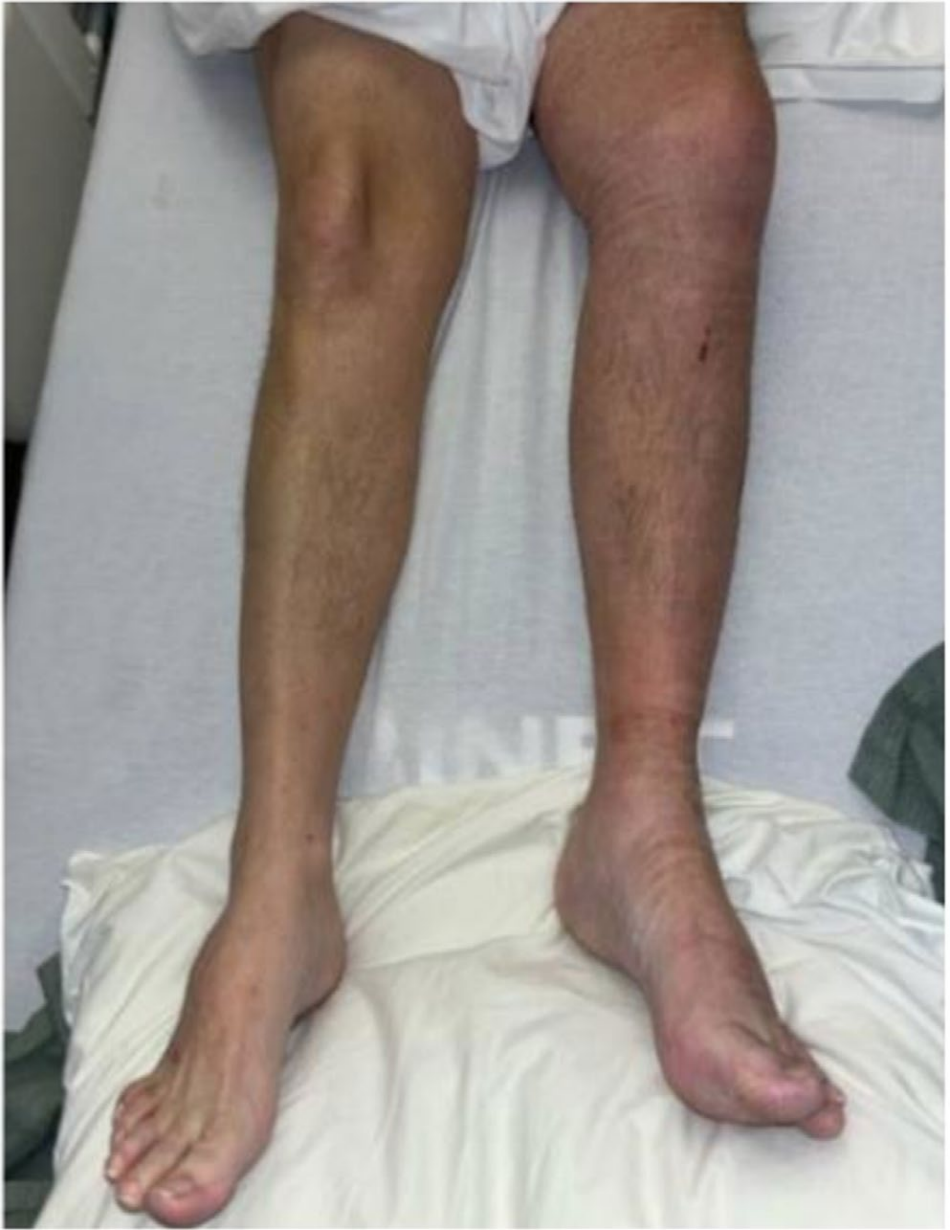
Day 3 of hospitalization, the patient's left lower extremity appears less cyanotic and discolored, with significant improvement in swelling.

By day 3, his leg showed reduced swelling and improved color (Figure 4). Given his clinical improvement, mechanical thrombectomy was deferred. He was transitioned to apixaban. Per the AMPLIFY trial, standard dosing for acute DVT is 10 mg twice daily for 7 days followed by 5 mg twice daily [3]. In this patient, anticoagulation was initiated at 5 mg twice daily, skipping the loading dose, to minimize hemorrhagic risk. He was discharged after one week with apixaban 5 mg BID, physical therapy, and hematology follow‐up. Plans included CT venography to assess filter patency, as IVC filters without anticoagulation are at risk for in‐filter thrombosis and recurrent DVT [4, 5].

## 5 Discussion

The clinical presentation and demographics of this case were consistent with subjects in previous literature, including being male, age older than sixty, and involvement of the left lower extremity [6]. Despite the severity of this patient's DVT in terms of massive size and venous obstruction, the healthcare team in this setting was able to avoid common poor outcomes of PCD, including venous gangrene formation, amputation, and death [7]. 30% of patients with PCD have a simultaneous PE, consistent with this case report [8]. The overall rate of mortality of PCD is estimated to range from 20% to 40% and the risk of amputation from 20% to 50% [2]. Although malignancy is the most common etiology of PCD (20%–40%), the patient in this case study had many other risk factors for development of PCD, including long smoking history, immobility, and IVC filter placement [2].

Definitive treatment of PCD includes either anticoagulation, catheter‐directed thrombolysis, and mechanical thrombectomy or a combination of these interventions depending on patient severity and comorbidity. Recent studies have shown that mechanical thrombectomy has improved vein patency [9]. A trend toward minimally invasive venous interventions such as endogenous radiofrequency ablation for varicose veins may suggest an overall inclination toward vascular surgery interventions [10]. However, mechanical thrombectomy does have a risk of vein damage and a prospective study displayed an 8.6% serious adverse event incidence after this intervention [11]. A case report describing mechanical thrombectomy for PCD noted that the patient suffered an ischemic stroke days post‐procedure, highlighting the risk of paradoxical embolism. In the setting of repairing arterial aneurysms, endovascular interventions possess exceedingly difficult‐to‐manage complications such as type II endovascular leaks [12]. Mechanical thrombectomy was not pursued because of the additional thrombus in the pulmonary vasculature that would not be addressed. If PCD was not resolved with systemic anticoagulation, mechanical thrombectomy may have been considered as the next intervention. Catheter‐directed thrombolysis has increased early major bleeding compared with anticoagulation alone, making this the least appropriate intervention in the setting of this case [13].

There are limited studies regarding PCD management after a recent hemorrhagic stroke. A published case report described the management of a patient with PCD who sustained a recent gastrointestinal bleeding [14]. Clinicians employed a modified anticoagulation regimen similar to ours, achieving successful management of PCD without recurrence of the bleed. Guideline reviews suggest that restarting anticoagulation after ischemic stroke and after stabilization of a hemorrhagic stroke reduces thromboembolism and mortality without a disproportionate rise in recurrent ICH when timing is tailored to infarct size/hemorrhagic risk and guided by repeat neuroimaging [15, 16].

As highlighted by the RIVAS study, there remains significant variability in the adoption of DOACs in vascular surgery practice, with many clinicians underutilizing them despite evidence of efficacy [17]. In this case study, anticoagulation was decided as the best option rather than thrombectomy and direct catheter lysis because of his additional PE in the right interlobar pulmonary artery that would not be addressed solely by mechanical thrombectomy as well as the increased risk of brain bleeding when using catheter‐directed alteplase.

The systemic anticoagulation was tailored to a safer profile and was monitored with consistent neurological checks and CT of the head. The 2020 American Society of Hematology VTE guidelines approve consideration of a lower‐dose direct oral anticoagulant regimen in patients at risk of intracranial bleeding [18]. Furthermore, pharmacokinetic data show that 5 mg twice daily yields less than half the systematic drug exposure of implementing the 10 mg twice daily loading dose regimen [19].

Despite mitigating risks of bleeding inherent to DOACs, the risks of intracranial hemorrhage remain. The incidence of intracranial hemorrhage after starting anticoagulation is estimated to be 1% [13]. Introducing anticoagulation after ischemic stroke has a hemorrhagic conversion rate of 2%–6% [18]. The ischemic stroke the patient sustained was not a large vessel occlusion, making the location for a hemorrhagic conversion relatively less compromising. The noncontrast CT head did not show an acute change as well, further developing a case to begin systemic anticoagulation.

**FIGURE 5:**
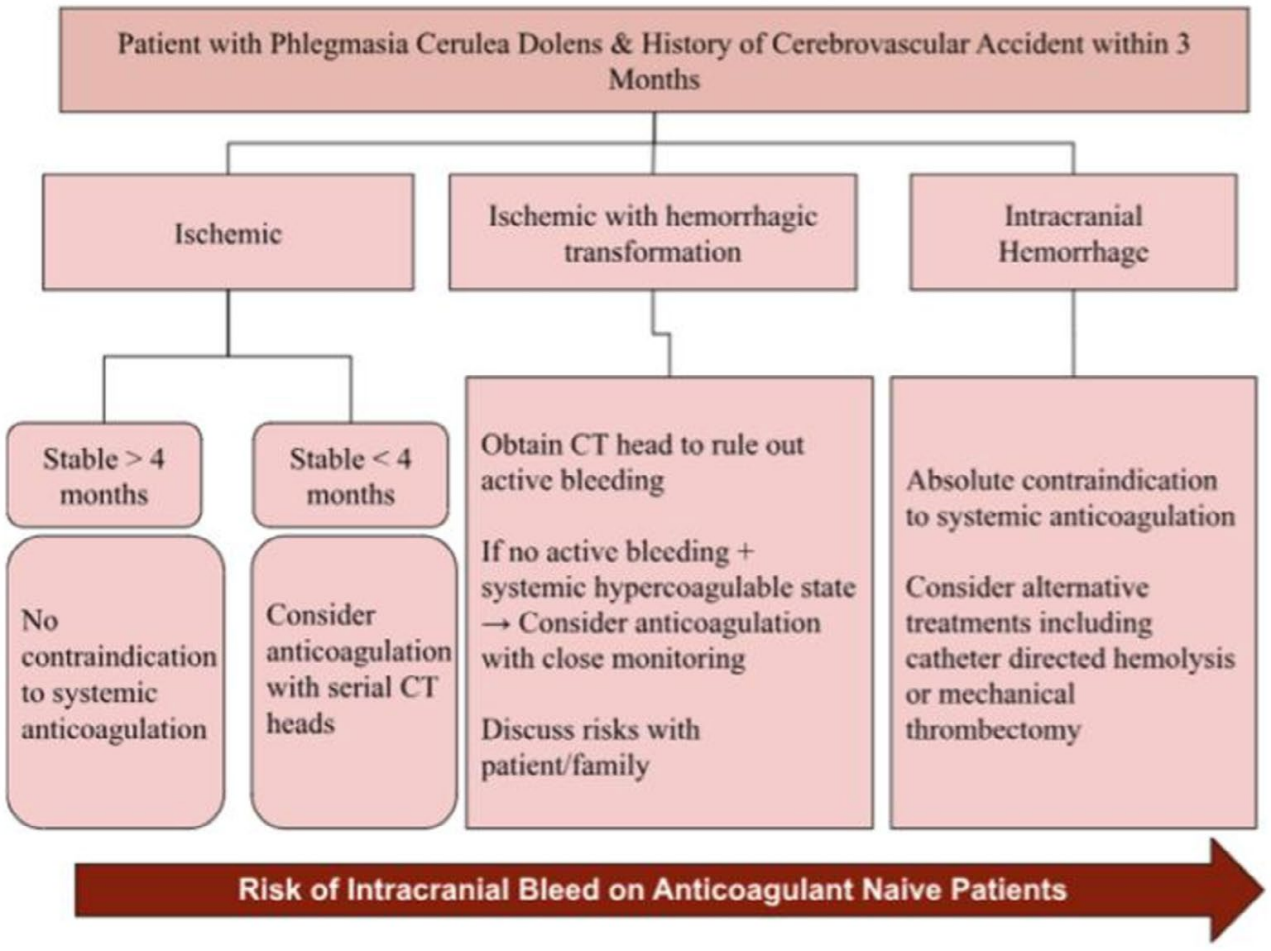
Proposed management flowchart for patients with PCD and recent cerebrovascular accidents. This algorithm highlights the role of serial CT imaging and multidisciplinary input in guiding whether systemic anticoagulation is safe to pursue.

A flowchart was devised to propose management of patients with PCD with recent cerebrovascular accidents (Figure 5). This algorithm may be applied when treating PCD in patients with recent cerebrovascular accidents to guide whether systemic anticoagulation is safe. It highlights the importance of serial CT imaging and multidisciplinary input. To use, first determine if the patient sustained a cerebrovascular accident within the past three months. In cases of ischemic stroke without hemorrhagic transformation that occurred more than four months ago, systemic anticoagulation is generally considered safe [20]. If the ischemic stroke occurred within the past four months, anticoagulation may still be considered provided that serial head CT monitoring is performed [20]. For ischemic strokes complicated by hemorrhagic transformation, anticoagulation can be cautiously pursued only after imaging confirms no active bleeding and the patient/family is counseled about hemorrhage risks. Greater than four weeks since cerebral hemorrhage is reassuring to resume anticoagulation [20]. In cases of primary intracranial hemorrhage, anticoagulation is contraindicated due to potential hematoma expansion and alternative interventions may be prioritized [21].

## 6 Conclusion

Management of PCD in the setting of a recent cerebrovascular accident requires a timely risk‐to‐benefit discussion. The patient in this case report was able to receive systemic anticoagulation treatment for PCD despite a recent hemorrhagic stroke through a tailored anticoagulation regimen, serial CT head non‐contrast, and neurological checks. Our report highlights the importance of individualized, multidisciplinary approaches to balancing thrombosis and bleeding risks in complex patients. This case underscores the limited therapeutic options for patients with recent ICH who remain at high risk of progressive venous thrombosis despite IVC filter placement. Additional research is needed to guide optimal management strategies in this high‐risk population.

## Author Contributions

**Reem Sarsour:** writing – original draft, writing – review and editing. **Veerpal Sond:** conceptualization, supervision, writing – original draft, writing – review and editing. **Cameron Casillas:** writing – original draft. **Elizabeth Morrison‐Banks:** project administration, resources. **Niki Mohammadi:** conceptualization, supervision, writing – original draft, writing – review and editing.

## Consent

A written informed consent was obtained from the patient to publish this report in accordance with the journal's patient consent policy.

## Conflicts of Interest

The authors declare no conflicts of interest.

## Data Availability

Patient consented for permission to draft a case report. If additional data regarding the case are requested, it can be made available from the corresponding author upon reasonable request. Due to patient confidentiality and ethical considerations, detailed clinical data and imaging beyond what is included in the manuscript cannot be shared publicly.
